# Identification and functional characterization of intermediate-size non-coding RNAs in maize

**DOI:** 10.1186/s12864-018-5103-1

**Published:** 2018-10-04

**Authors:** Dandan Li, Huili Qiao, Wujie Qiu, Xin Xu, Tiemei Liu, Qianling Jiang, Renyi Liu, Zhujin Jiao, Kun Zhang, Lijun Bi, Runsheng Chen, Yunchao Kan

**Affiliations:** 1 China-UK-NYNU-RRes Joint Laboratory of insect biology, Henan Key Laboratory of Insect Biology in Funiu Mountain, Nanyang Normal University, 1638 Wolong Road, Nanyang, 473061 Henan China; 2 Center for Agroforestry Mega Data Science and FAFU-UCR Joint Center for Horticultural Biology and Metabolomics, Haixia Institute of Science and Technology, Fujian Agriculture and Forestry University, Fuzhou, 350002 China; 3 Bioinformatics Laboratory and National Laboratory of Biomacromolecules, Institute of Biophysics, Chinese Academy of Sciences, Beijing, 100101 China

**Keywords:** Maize, Intermediate-size ncRNAs, Stress

## Abstract

**Background:**

The majority of eukaryote genomes can be actively transcribed into non-coding RNAs (ncRNAs), which are functionally important in development and evolution. In the study of maize, an important crop for both humans and animals, aside from microRNAs and long non-coding RNAs, few studies have been conducted on intermediate-size ncRNAs.

**Results:**

We constructed a homogenized cDNA library of 50–500 nt RNAs in the maize inbred line Chang 7–2. Sequencing revealed 169 ncRNAs, which contained 58 known and 111 novel ncRNAs (including 70 snoRNAs, 27 snRNAs, 13 unclassified ncRNAs and one tRNA). Forty of the novel ncRNAs were specific to the Panicoideae, and 24% of them are located on sense-strand of the 5′ or 3′ terminus of protein coding genes on chromosome. Target site analysis found that 22 snoRNAs can guide to 38 2’-*O*-methylation and pseudouridylation modification sites of ribosomal RNAs and small nuclear RNAs. Expression analysis showed that 43 ncRNAs exhibited significantly altered expression in different tissues or developmental stages of maize seedlings, eight ncRNAs had tissue-specific expression and five ncRNAs were strictly accumulated in the early stage of leaf development. Further analysis showed that 3 of the 5 stage-specific ncRNAs (Zm-3, Zm-18, and Zm-73) can be highly induced under drought and salt stress, while one snoRNA Zm-8 can be repressed under PEG-simulated drought condition.

**Conclusions:**

We provided a genome-wide identification and functional analysis of ncRNAs with a size range of 50–500 nt in maize. 111 novel ncRNAs were cloned and 40 ncRNAs were determined to be specific to Panicoideae. 43 ncRNAs changed significantly during maize development, three ncRNAs can be strongly induced under drought and salt stress, suggesting their roles in maize stress response. This work set a foundation for further study of intermediate-size ncRNAs in maize.

**Electronic supplementary material:**

The online version of this article (10.1186/s12864-018-5103-1) contains supplementary material, which is available to authorized users.

## Background

With more and more genomes being sequenced, numerous non-coding RNAs (ncRNAs) have been identified, their functions are also being revealed [1–8]. In maize, a large number of miRNAs have been identified in different lines and developmental conditions [9–12], such as miR159, miR164, miR167, miR393, miR408 and miR528 are mainly involved in root development and stress responses [13, 14]. miR160, miR167, miR164, miR169 and miR393 can respond to hormone signaling [14]. Genome-wide identification of long non-coding RNAs (lncRNAs) was also accomplished in maize, more than 40, 000 lncRNAs have been identified, most of them were expressed in tissue-specific manner [15–20]. Some lncRNA can participate in maize pollen development, such as lncRNA *Zm401*, knocking down of which can significantly affect three key genes for pollen development (*ZmMADS2*, *MZm3–3*, and *AmC5*), then result in male sterility [21]. Other lncRNAs can response to stress conditions, Wang et al. found some transposable-element-derived long intergenic non-coding RNAs (TE-lincRNAs) can be induced or inhibited by cold, heat, drought, salt or highlight stress in rice and maize [22]. However, studies focused on the identification and functional characterization of intermediate-size ncRNAs (50–500 nt) in maize is rare. Small nucleolar RNAs (snoRNAs) and small nuclear RNAs (snRNAs) are the major classes of intermediate-size ncRNAs, snoRNAs can guide site-specific RNA modifications of ribosomal RNAs (rRNAs), snRNAs, tRNAs as well as mRNAs [23], while snRNAs can participate in alternative splicing in mRNA processing. Nowadays, more and more evidence showed that snoRNA, the old dog, has new tricks [24]. Through sRNA deep sequencing studies, several reports have identified snoRNA-derived small RNAs (sdRNAs) [25–29], which broaden the roles of snoRNAs. sdRNAs in animals are preferentially derived from the 3′ end of H/ACA snoRNAs and the 5′ end of C/D snoRNAs [29–31]. While sdRNAs of Arabidopsis are always associated with AGO7 [30]. Recently, a snoRNA-derived piRNA piR30840 was found to be accumulated in human CD4 primary T-lymphocytes, piR30840 can bind to the intron of interleukin-4 through AGO4/PIWI14/piRNA complex, then lead to the pre-messenger RNA (pre-mRNA) decay of interleukin-4 [32]. Meanwhile, snoRNAs can be the biomarkers for diseases and physiological changes [33, 34], such as SNORA21 and SNORD126, can be accumulated more in colorectal adenomas and hepatocellular carcinoma, respectively [35, 36]. Moreover, some snoRNAs were found to play roles in pre-mRNA alternative splicing [24, 37–42], suggesting that the potential roles of snoRNAs are just beginning to be appreciated.

Maize (*Zea mays* L.) is one of the most important food crops in the world. The maize genome has been sequenced, and nearly 85% of the 3.2 GB genome sequence is composed of hundreds of families of transposable elements [43]. Because the vast majority of the maize genome consists of non-protein-coding regions, there is great potential for discovering more ncRNAs in maize.

In this work, we constructed a cDNA library of 50–500 nt RNAs that were extracted and size-selected from seedling and developing grain total RNAs of the maize inbred line Chang 7–2. From 1,273 full-length cDNA sequences, we identified 111 novel ncRNA candidates. The expression pattern of these ncRNAs from different tissues and different developmental stages were studied. Functional exploration of ncRNAs was also done under drought and salt stress. Our results provide the first genome-wide survey and functional characterization of intermediate-size ncRNAs in maize.

## Results

### Identification of intermediate-size ncRNAs in maize

**Fig. 1:**
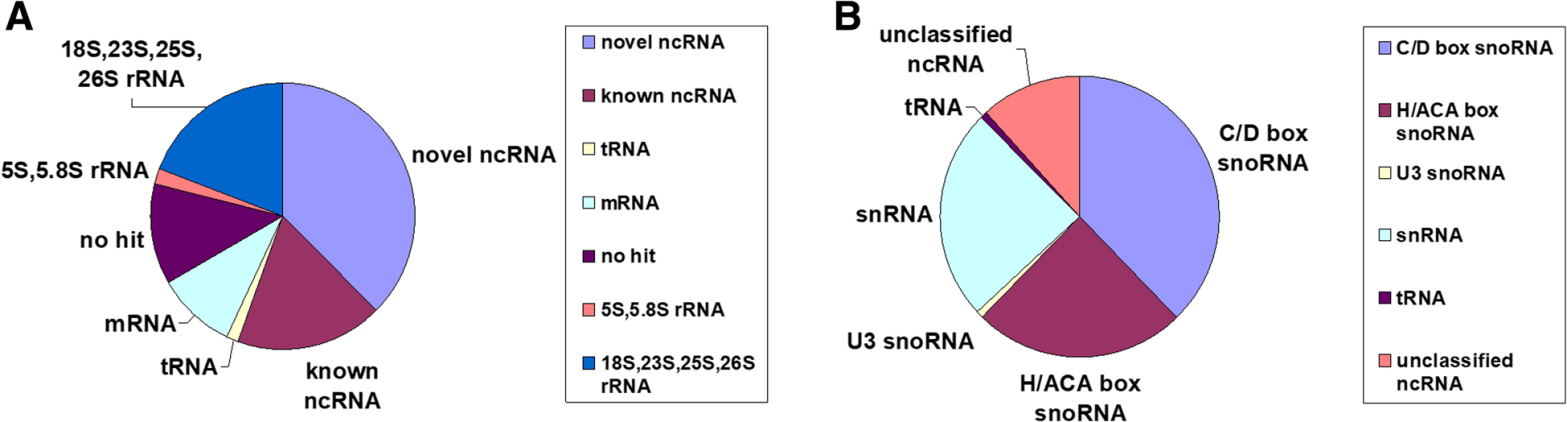
Classification of sequenced full-length cDNAs (**a**) and novel ncRNA candidates (**b**)

To identify new ncRNA candidates in maize, we constructed a full-length intermediate-size ncRNA-enriched library (50–500 nt) from the maize (*Zea mays* L.) inbred line Chang 7–2, the wide-spread planted line in Henan province of China. Total RNAs were isolated from seven tissues harvested at eleven developmental stages of maize seedlings and five stages of developing grains. In order to obtain the 50–500 nt RNA fragments precisely and to remove the known RNAs with high abundance, cDNA library was constructed with the method of Deng et al. but not RNA-seq [44, 45]. Probes with poly (A) tails were used to remove the enriched RNAs such as mRNAs, rRNAs, and snRNAs to homogenize the library, then 50–500 nt RNAs were sliced from the gel and inserted into the pGEM-T vector for electro-transformation. Approximately 5,000 clones were picked out and tested by PCR, the products were identified by 6% Polyacrylamide gel electrophoresis (PAGE) and selected by their length. Totally, 1,273 clones were picked out and sequenced. After removing duplications with same sequence, 297 unique sequences were identified, among which 63 transcripts (21%) were rRNAs, 169 transcripts (57%) were annotated as other ncRNAs, the rest were error reads or mRNA degradation products (Fig. 1a), which indicated that the probes used to fish out rRNAs was not so efficient, but the yield of other ncRNAs in unique transcripts was high, accomplished 57%.

Of the 169 ncRNAs, 58 were known ncRNAs, which contained 42 snRNAs, 7 snoRNAs, 5 Signal recognition particle RNAs (SRPs) and 4 tRNAs. Meanwhile, 111 (66%) transcripts were identified as novel ncRNA candidates. To verify these ncRNAs were real transcripts but not degradation products, the expression of 84 randomly selected ncRNAs were identified by northern blots (data in the ncRNA expression results). In addition, full length of 14 ncRNAs was checked by 5′ and 3’ RACE. Results showed besides the RACE sequences differed from the corresponding cDNA sequences by at most one to two terminal nucleotides, the other results showed that these ncRNA sequences are indeed full-length transcripts.

Based on structural features and sequence similarity to known ncRNAs of other species (NONCODE version 5.0), 111 novel ncRNAs were classified into different categories. Seventy of them (63%) were annotated as snoRNA-like transcripts, including 42 C/D box, 27 H/ACA box snoRNAs, and one U3 snoRNA. Twenty-seven ncRNAs (24%) were classified as snRNAs and one as tRNA. The other 13 ncRNA candidates (12%) could not be assigned to any group due to lack of known motifs or secondary structure hallmarks and are referred to as “unclassified ncRNAs” (Fig. 1b). Comparing to the RNA-seq method used in rice, which got 125 novel snoRNAs (9%), 59 (4%) snRNAs and a large number of unclassified ncRNAs (781) [45], we got high percentage of novel snoRNAs but low ratio of unclassified ncRNAs, which indicated that our library covered the main part of ncRNAs like snoRNAs and snRNAs in this length range.

### Conservation analysis shows a large number of Panicoideae-specific ncRNAs

To investigate whether the sequenced ncRNAs were conserved in flowering plants, we used the BLASTN program to search for their homologs in sorghum, rice, and *Arabidopsis* genomes. Results showed that 76 of the 169 unique ncRNAs had counterparts in the three plant genomes, including 3 snRNA families (57 unique snRNAs), 8 snoRNAs, 4 SRPs, 5 tRNAs, and 2 unclassified ncRNAs (Zm-79 and Zm-80). Almost all of the ncRNAs in maize (166 of 169 transcripts) had counterparts in the closely-related *Sorghum bicolor* genome (with identity more than 90%), among which 100 ncRNAs match the sorghum EST sequences, indicating that they are also expressed in sorghum. We also found 128 (76%) ncRNAs had counterparts in the rice genome (with identity more than 95%). The remaining 41 ncRNAs with little conservation were snoRNAs and unclassified ncRNAs (Additional file 1).

Targets predication showed that 16 C/D box and 6 H/ACA box snoRNAs could guide to 30 2’-O-methylation and 8 pseudouridylation sites of maize rRNAs and snRNAs, respectively. After removing the conserved ncRNAs and transcripts with conserved functional elements, 40 ncRNAs (containing 9 C/D box, 18 H/ACA box snoRNAs and 13 unclassified ncRNAs) still could not identify any homolog or ortholog to *Arabidopsis* or rice, indicating that they were specific to Panicoideae.

### Genomic organization of maize ncRNAs

We compared the genomic locations of the novel ncRNAs with annotated protein-coding genes in the maize genome. Results showed that more than half of novel ncRNAs were located in the intergenic regions and 15% in introns (Additional file 2: Table S1), 70% host genes of the intronic ncRNAs encode ribosomal proteins (Additional file 2: Figure S1), which is similar to other organisms [23]. Twenty-seven novel ncRNAs (24% of total novel ncRNAs) had overlaps with the 5′ or 3′ untranslated region (UTR) of protein coding genes on the sense strand (Additional file 2: Table S1). This is similar to rice, in which more than half of the intermediate-size ncRNAs were located around the plus-strand of the 5′ and 3′ terminus of the coding sequences [45]. However, different to rice (in which the number of ncRNAs on the 3’ UTR is nearly two-fold as that on the 5’ UTR), 67% of the UTR-origin ncRNAs in maize are located on the 5’ UTR, and 78% of the UTR-origin ncRNAs are snoRNAs, but most of them have no predicated targets to rRNAs or snRNAs, which indicated that they might be orphan snoRNAs.

To verify that UTR-origin ncRNAs are not degradation products of protein-coding genes, 21 of the 27 UTR-origin ncRNAs were detected by the northern blot. Results showed that all of them can be hybridized with the correct size, but the corresponding host genes could not be detected (Additional file 2: Figure S2A and B), suggesting that the UTR-origin ncRNAs were full-length transcripts rather than degradation products of host genes. Moreover, 93% of the host genes of UTR-origin ncRNAs are annotated as hypothetical or pseudogenes (Additional file 2: Figure S3). To study whether these UTR-origin snoRNAs has the potential to be precursors of miRNAs, online software miRNAFold was used. Results showed that these snoRNAs had no potential to be miRNA precursors.

The ncRNAs that originated from Open Reading Frames (ORFs) had been found in other organisms, such as in *Drosophila* and rice [45, 46]. In maize, nine ncRNAs (8 U1 snRNAs variants and 1 C/D box snoRNA) were found to be located in ORFs. The host genes of the 8 snRNAs are annotated as pseudogenes with cDNA length between 141 nt and 400 nt. Northern blot results showed that only the U1 snRNA but not the host pseudogene had hybridization signals (Additional file 2: Figure S2D), indicating that the host pseudogenes might not be transcribed to coding RNAs.

In plants, most snoRNAs are found in polycistronic clusters. We examined the relative locations of intermediate-size ncRNAs in maize and found that 82 ncRNA variants (58 unique ncRNAs) formed 28 clusters on chromosomes (Additional file 1), 71% of which were snoRNAs. In contrast to rice, in which all snoRNA clusters are in intron and intergenic regions, there are only five intronic and three intergenic snoRNA clusters in maize, and the other 11 snoRNA clusters are located in the UTR regions of protein-coding genes (Additional file 1).

In rice, half of the snoRNA gene families expanded in the genome through tandem duplications [45]. In maize, many less tandem duplications were involved in the origination of ncRNAs. Only one C/D box snoRNA, Zm-19, which was transcribed from the second intron of gene GRMZM2G047727-T01, has five tandem repeats with an interval of 138 nt. The counterparts of these tandem repeats are also observed in the sorghum and rice genomes, but with different repeat numbers. Another two unclassified ncRNAs, Zm-79 and Zm-80, have nine tandem repeats on six chromosomes (chromosome 1, 2, 3, 5, 7, and 10) with intervals between 2791 bp and 2822 bp. They also have counterparts of tandem repeats in the sorghum, rice, and *Arabidopsis* genomes with less repeat numbers, indicating that these tandem repeats are highly conserved between monocots and dicots.

### ncRNAs with tissue-specific expression

**Fig. 2:**
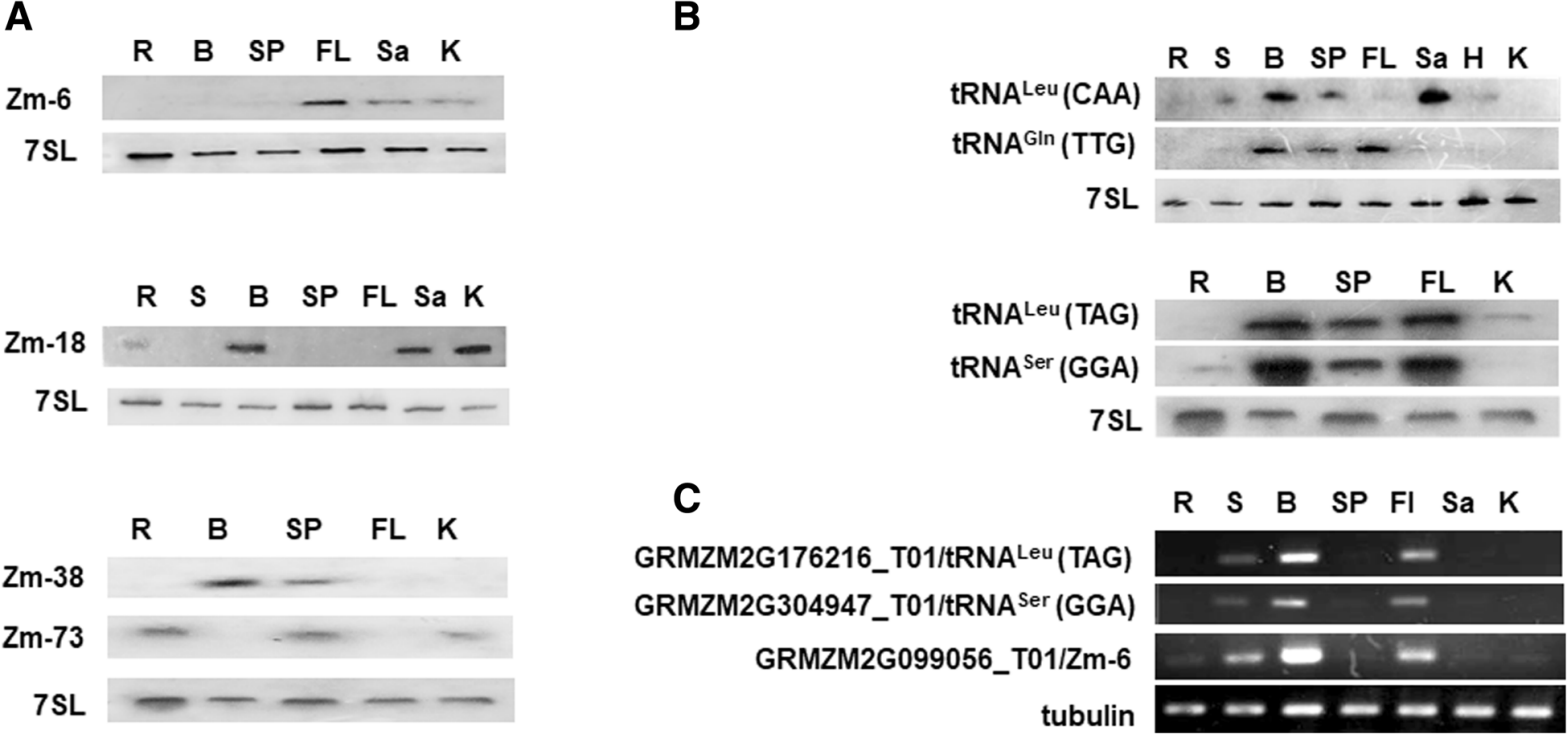
Expression pattern of tissue-specific ncRNAs and their neighboring genes by Northern blot and semi-quantitative RT-PCR. (**a**) Expression pattern of three C/D box snoRNAs (Zm-6, Zm-18, and Zm-38) and one unclassified ncRNA (Zm-73) in different tissues of maize by northern blot. (**b**) Expression pattern of four tRNAs in different tissues of maize by northern blot. (**c**) Expression pattern of the neighboring genes of Zm-6, tRNA^Leu^ (TAG), and tRNA^Ser^ (GGA) in different tissues of maize by semi-quantitative RT-PCR. R, S, B, SP, FL, Sa, H, and K representing the root, stem, blade, sheath & petiole, flag leaf, stem apex, hypocotyl, and developing kernel, respectively. 7SL was used as an internal control for ncRNAs (**a**) and (**b**). The *tubulin* gene was used as an internal control for neighboring genes (**c**)

**Fig. 3:**
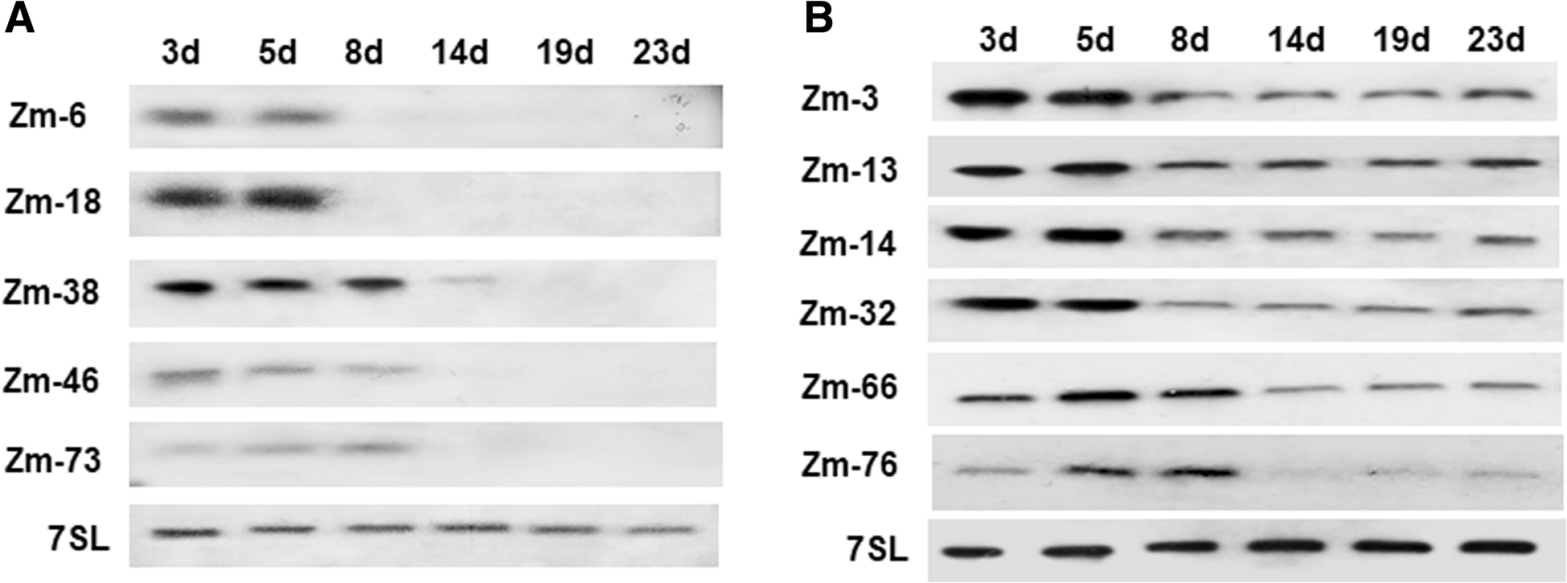
Expression pattern of ncRNAs specific (**a**) and enriched (**b**) in the early stage of leaf development by northern blot. 3d, 5d, 8d, 14d, 19d, 23d represent RNAs extracted from leaves at 3, 5, 8, 14, 19, and 23 DAG, respectively. 7SL was used as an internal control

The ncRNAs are frequently expressed in specific tissues or developmental stages in eukaryotes, indicating their diverse roles in various cellular processes [42, 47–50]. We used northern blots to compare the expression of the intermediate-size ncRNAs in different tissues and developmental stages of maize. The expression of 64 unique novel ncRNAs and 20 known ncRNAs were detected, results showed that 43 ncRNAs had significantly altered expression (Figs. 2, 3, Additional file 2: Figures S2A, B, D and S5A and S6A). These include 31 snoRNAs (20 C/D box and 11 H/ACA box snoRNAs), 4 unclassified ncRNAs, 4 snRNAs (three U2 and one U5 snRNA), and four tRNAs. Nearly 65% of the differentially-expressed snoRNAs had no predicted targets and might be orphan snoRNAs.

Most of the intronic and UTR-origin ncRNAs exhibited a similar expression pattern with their host genes in different tissues (Additional file 2: Figure S2 and S5). However, the expression of intergenic ncRNAs had various correlations to their neighboring genes, consistent in some tissues (root, blade, stem, flag leaf and developing kernel), but different in other tissues (sheath & petiole and stem apex) (Additional file 2: Figure S6), indicating their different regulatory roles in various organs. Interestingly, Zm-46 had two transcript isoforms in the blade and developing kernel (Additional file 2: Figure S5), indicating that alternative splicing products were generated in different tissues.

Eight ncRNAs exhibited tissue-differential expression (Fig. 2), including three C/D box snoRNAs (Zm-6, Zm-18, and Zm-38), one unclassified ncRNA, and four tRNAs. Such as Zm-6, had more expression in the flag leaf, stem apex, and developing kernel, but little or no detectable expression in the blade and root (Fig. 2a); however, its antisense upstream gene GRMZM2G099056-T01(*roothairless*) (genomic position was shown in Additional file 2: Figure S4) accumulated more in the blade and root (Fig. 2c), indicating that Zm-6 might play reverse role in the regulation of *roothairless* during maize root development.

All four tRNAs with altered expressions were accumulated more in leaves (Fig. 2b). To further study the functions of these tRNAs, we analyzed their genomic organization and the expression pattern of their neighboring genes. We found that each tRNA had multiple copies in the genome. For example, there are 25 copies of tRNA^Ser^ (GGA) in the maize genome, and all of them are located on the antisense strand of LTR/Copia retrotransposons. The expression of tRNAs were positively correlated with their neighboring genes (Fig. 2b and c), indicating that the tissue-differential expressed tRNAs might function together with their neighboring genes in the leaf development of maize.

### ncRNAs accumulated more in juvenile stage of maize seedling

The change of gene expression in leaves during the seedling stage is important for grain yield. During this period, leaves exhibit photomorphogenic growth and accumulate much more nutriment in preparation for the reproductive stage. With Northern blots analysis, we found that 11 ncRNAs can accumulated more in the early stages of leaf development (Fig. 3), including 2 unclassified ncRNAs and 9 snoRNAs. Furthermore, five ncRNAs (Zm-6, Zm-18, Zm-38, Zm-46, and Zm-73) were strictly expressed before 14 DAG (Fig. 3a). Further analysis showed that besides Zm-38, the other snoRNAs have no predicated targets to rRNAs and snRNAs, which appeared to be orphan snoRNAs. From 8 to 11 DAG, maize enters into the trefoil stage, the seedling begins transition from juvenile to adult, a lot of protein-coding genes had differential expressions before and after the conversion, such as *Teopod* genes, the accumulation of *Tp*1, *Tp*2, and *Tp*3 before the transition can promote juvenility of maize seedling [51, 52]. In our results, the accumulation of snoRNAs in leaves before the 14th day of seedling indicated their roles in juvenile maintainence of maize.

### Stress-regulated ncRNAs

**Fig. 4:**
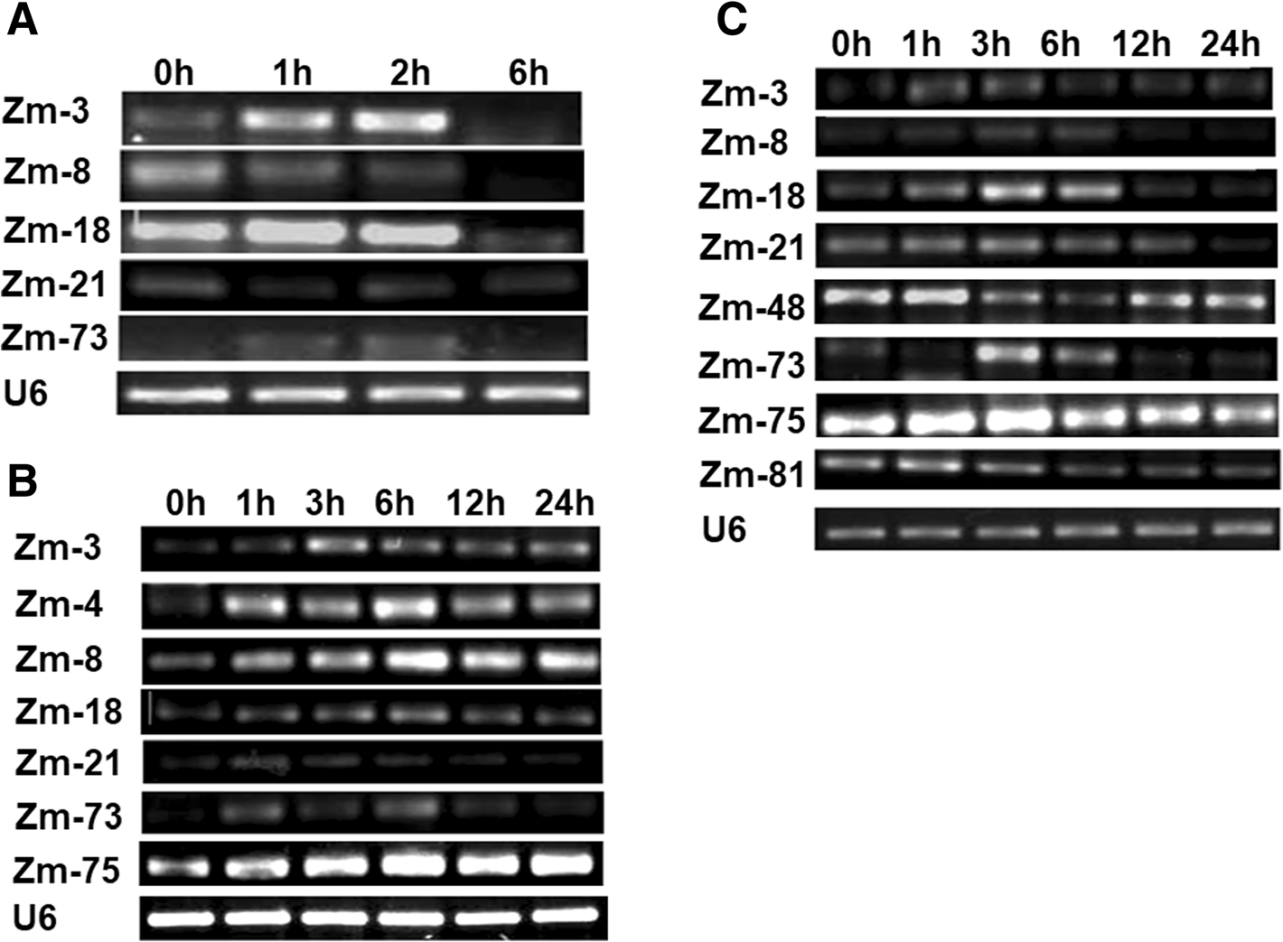
Semi-quantitative RT-PCR results of ncRNA genes under PEG6000 simulated-drought stress (**a**), ABA simulated-drought stress (**b**), and NaCl simulated-salt stress (**c**). 0, 1, 3, 6, 12, and 24 h represented RNAs extracted from roots at the 0, 1, 3, 6, 12, and 24 h after different abiotic stress. U6 was used as an internal control

Accumulated evidence supports important roles for ncRNAs in plant response and adaptation to abiotic stresses [53]. To investigate whether intermediate-size ncRNAs are regulated by abiotic stresses, we used polyethylene glycol 6000 (PEG6000), abscisic acid (ABA) and NaCl to simulate drought and salt stress to study the expression of ncRNAs in maize roots. Results showed that 3 ncRNAs (Zm-3, Zm-18, and Zm-73) were induced strongly at the early stage of PEG simulated drought stress (1 h), but their expression decreased sharply after 6 h treatment (Fig. 4a), a similar result was also found under ABA stress (Fig. 4b). Moreover, Zm-18 and Zm-73 can be induced strongly at 3 h under salt stress, and Zm-3 was induced quickly after 1 h NaCl treatment (Fig. 4c), which indicated their roles in stress response.

However, other ncRNAs can be inhibited by stress, like Zm-8, whose expression was decreased after 2 h of PEG-simulated drought stress (Fig. 4a). But which interesting is the expression of Zm-8 can be induced after 6 h ABA treatment (Fig. 4b), which indicated Zm-8 might participate in different signal pathways in different treatments although PEG and ABA both simulated the drought stress.

## Discussion

### Panicoideae-specific ncRNAs

Maize and sorghum are both Panicoideae plant, maize genome is three times larger than the sorghum genome, but 93% of their protein coding gene families were conserved [54, 55]. In our results, almost all the ncRNAs detected in maize can find counterparts on sorghum genome, and 100 ncRNAs can blast to the EST sequences of sorghum, which indicated that ncRNAs of same subfamily were also highly conserved.

But when comparing the ncRNAs between monocot and dicot, we found 40 ncRNAs were specific to Panicoideae, with more diversity to rice and wheat. Moreover, nearly half of them were located in intergenic regions. Divergency of Monocot and dicot was taken placed around 200 mya ago [56, 57], during the last ~ 3 million years, the size of the maize genome has expanded to 2.3 GB via a proliferation of long terminal repeat retrotransposons (LTR retrotransposons) in the intergenic region [43, 58–63]. The large number of Panicoideae-specific ncRNAs being located in the intergenic region of maize genome indicated that these ncRNAs might also experience the selective pressure during maize evolution.

### Pseudogene-derived ncRNAs

The distribution of maize-novel ncRNAs on chromosomes was varied, 51% loci were intergenic and 15% were in introns. Twenty-four percent of the novel ncRNAs originated from the 5′ or 3’ UTR of protein coding genes, moreover, a large number of their host genes are annotated as pseudogenes. Pseudogenes are complete or partial of protein-coding genes with no functional protein products [64, 65]. For the high conservation between pseudogenes and their parental protein-coding genes, some novel function of pseudogenes were verified [66]. Guo et al. found 145 pseudogene-derived siRNAs in rice genome, they can interact with their antisense RNAs (38 cases) or form double-strand RNAs with their adjacent pseudogenes (2 cases), some of them are abundant in specific rice developmental stages or physiological growth conditions, suggesting their potential roles in rice development [67]. Recent studies also established essential roles of pseudogene-derived lncRNAs in development and disease in mammalian [68–71]. Like the pseudogene-derived long non-coding RNA DUXAP10, can bind to histone demethylase lysine-specific demethylase 1 and silence the expression of *p21* and phosphatase and tensin homolog (*PTEN*), then promote colorectal cancer cell growth [70]. In our results, 25 novel ncRNAs were originated from the 5′ or 3’ UTR of hypothetical or pseudogenes, many of them had tissue differential expression. Further analysis showed these pseudogene-derived ncRNAs had no potential to be precursor of miRNAs or siRNAs of maize, which indicated that they might play roles by themselves. Whether they can function as reverse regulator to the parental protein-coding genes of pseudogenes and how they play roles in maize development need much more evidence.

### Expressions of tRNAs showed codon usage bias in leaves

In our results, four tRNAs had tissue-differential expression, which was similar to that have been found in other organisms. Like in human brain, nuclear encoded tRNAs had a high expression level than in other tissues [72]. In four sequenced cotton species, Wang et al. found that the pyrimidine-rich codons were used much more than purine-rich codons [73]. Tissue-specific expression of different tRNA species indicate that codon bias might play important roles in different tissues and developmental stages of organisms [72]. We found the expression of tissue-specific tRNAs accumulated more in the leaf organs of maize, with less or none in the root, meristem, and developing kernel. Leaf-related organs are the most productive parts for protein biogenesis, in which tRNAs preferred the codons of Leu, Gln, and Ser, the uncharged amino acids, which indicated the existence of codon bias in maize leave development.

### Stress-induced intermediate-size ncRNAs of maize

Plants are exposed to ever-changing environmental conditions including drought, freezing, and salinity. Modulation of gene expression is the key method for plants to respond, adjust and adapt to stress conditions. Besides protein-coding genes, non-coding RNAs also play essential roles in this process, such as miRNAs, trans-acting siRNAs, heterochromatic siRNAs as well as long non-coding RNAs. Molecular mechanisms of miRNAs and siRNAs in stress conditions have been well-studied [16, 74–77]. Functional roles of lncRNA in plant are gradually revealed, such as acting as ceRNAs to block the interaction between miRNAs and their target genes [78], or through chromatin modification or DNA methylation (RdMD) with other proteins [16, 79–82], like the cold-induced lncRNAs, *COOLAIR* and *COLDAIR*, they can reduce the H3K36me3 or H3K4me2 level at *FLOWERING LOCUS C* (*FLC*) and recruit polycomb repressive complex 2 (PRC2) to promote H3K27me3 accumulation at *FLC* to inhibit the expression of *FLC*, and lead to the flowering repression [80–83]. When we used PEG6000, ABA, and NaCl to simulate the drought and salt stress, we found many ncRNAs can be induced. For example, Zm-3, Zm-18, and Zm-73 were strongly induced at the early stage of drought stress, and Zm-18 and Zm-73 were strongly induced at 3 h after NaCl treatment. The same phenomenon has been found in *Arabidopsis*, the lncRNA npc60 showed a 100-fold increase under salt treatment [84], which indicated their roles in stress response. Further analysis showed that Zm-3, Zm-18 and Zm-73 had no potential to be precursor or sponge to maize known miRNAs, so whether these snoRNAs function as scaffold of protein complex or affect histone modification need much more evidence.

## Conclusions

In this study, we provided a genome-wide identification and functional analysis of ncRNAs with a size range of 50–500 nt in maize. One-hundred and eleven novel ncRNAs were cloned and 40 ncRNAs were determined to be specific to Panicoideae. Target site analysis predicated a total of 38 2’-O-methylation and pseudouridylation modification sites of rRNAs and snRNAs. Furthermore, analysis of the expression profiles of the novel ncRNAs showed that 43 ncRNAs changed significantly during maize development, three ncRNAs can be strongly induced under drought and salt stress, suggesting their roles in maize stress response.

## Methods

### Construction of the cDNA library of 50–500 nt ncRNAs in maize

To construct a full-length intermediate-size (50–500 nt) ncRNA library of maize (*Zea mays* L.) inbred line Chang 7–2 (gift from Dr. Jihua Tang of Henan Agricultural University), total RNAs were isolated from seven tissues harvested at eleven developmental stages of maize seedlings and five stages of developing grains. First, the seeds of maize were surface-sterilized in 0.1% HgCl_2_ for 10 min and rinsed in distilled water 10 times, then soaked in the dark overnight and placed on filter papers soaked with distilled water for another 24 h. The germinated seeds were transferred into 25 cm seedling pot with vermiculite at a photoperiod of 16/8 h (light/darkness) and 25/15 °C with a relative humidity of 65% in phytotron, the light intensity was 200 μmol photons m^− 2^ s^− 1^. Tissues of root, stem, blade, sheath & petiole, flag leaf, stem apex and hypocotyl were collected from eleven developmental stages of maize seedlings, the 3, 5, 8, 11, 14, 17, 19, 23, 26, 34, and 42 days after germination (DAG). Meanwhile, maize inbred line Chang 7–2 was also planted at the farmland of Henan Agricultural University (Zhengzhou, China). Immature seeds were collected from 5, 7, 9, 14 and 19 days after artificial pollination (DAP). Samples were immediately frozen in liquid nitrogen. TRIzol (Thermo Fischer Scientific) was used for RNA extraction. Equal amount of RNA from 82 samples were mixed together to generate a homogenized library.

Small RNA library (50–500 nt) was constructed with the method of Deng et al. [44]. The small RNA fraction was isolated with a QIAGEN tip (QIAGEN) from 200 μg total RNAs. Messenger RNAs (mRNAs) and rRNAs were removed with the Ambion® Poly(A) Purist™ MAG (Thermo Fischer Scientific) and MICROB*Express*™ kits (Thermo Fisher Scientific) with the magnetic oligo(dT) cellulose, the probes used to fish out rRNAs were listed in Additional file 2: Table S2. RNAs were dephosphorylated with calf intestine alkaline phosphatase (Thermo Fisher Scientific) and then ligated to the 3-adaptor oligonucleotide (3 AD, with a restriction endonuclease site of SacI) by T4 RNA ligase (Thermo Fisher Scientific). The ligation product was split into two aliquots: one was treated with polynucleotide kinase (Thermo Fisher Scientific) for the uncapped RNA, and the other was treated with tobacco acid pyrophosphatase (Thermo Fisher Scientific) to remove 5′-end methyl-guanosine caps from capped RNA. The two parts of RNAs were ligated to the 5′-adaptor oligonucleotide (5 AD, with a restriction endonuclease site of KpnI). cDNA was prepared with Thermoscript™ reverse transcriptase (Thermo Fisher Scientific) at 50 °C, using oligo 3RT (in Additional file 2: Table S3) as the reverse transcription primer. The cDNA was PCR-amplified and digested with SphI and SacI (NEB) and cloned in pGEM-4Z vector (Promega). Transformation was performed with *E. coli* DH5α electrocompetent cells. Mono-clones were picked out and identified by PCR with 5CD (same DNA sequence with 5 AD) and 3RT (reverse complementary DNA sequence to 3 AD) primers (in Additional file 2: Table S3). PCR was performed at an annealing temperature of 55 °C with 20 cycles, PCR products were identified on 6% native PAGE, clones with same length of PCR product were reserved not more than 3, clones with different length of PCR product were picked out directly. Sanger sequencing method was used to identify different transcripts (Thermo Fisher Scientific).

### Bioinformatics analysis

mfold (version 4.6) was used to predict the secondary structures of RNAs [85]. MEME (version 4.9.1) was used to predicate the conserved box of ncRNAs [86]. Potential targets of snoRNAs were predicated by snoScan and snoGPS with the same criteria as Li et al. [87]. The *Zea mays* L. genome sequence and annotation data were downloaded from maizesequence.org (version 5b+), genome and EST sequences of *Oryza sativa* (version 7) and *Sorghum bicolor* (version JGI Sbi1) were download from PlantGDB, genome of *Arabidopsis thaliana* was download from TAIR (version 10). The homologs or orthologs of ncRNAs were identified with BLASTN (version 2.2.22+), the cutoff of sequence identity is 90% or 95%, the e-value is 1 × 10^− 10^. The online tool of miRNAFold was used to predicate whether ncRNAs could be precursors of miRNAs (http://evryrna.ibisc.univ-evry.fr/miRNAFold) [88], the parameters were default, species parameters were *Zea mays*.

### Northern blot and 5′- and 3′ - rapid amplification of cDNA end (RACE)

Northern blot was performed according the method of Li et al. [87]. RACE was performed as following, total RNAs were isolated from maize seedling with TRIzol method, RNAs were ligated to the 5′-adaptor (5 AD) and 3′-adaptor (3 AD) oligonucleotides with the method described above, cDNAs were reverse transcribed with Thermoscript™ reverse transcriptase (Thermo Fisher Scientific) at 50 °C, using oligo 3RT as the reverse transcription primer. Rapid amplification of cDNA ends (RACE) was performed by PCR amplification, with one primer specific to the ncRNA sequence and the other primer specific to the 5′-adaptor (5CD) or reverse complement to the 3′-adaptor (3RT) for 5′- or 3’-RACE, respectively. All primers were listed in Additional file 2: Table S3.

### Stress treatments of maize seedling

Maize seeds were surface-sterilized in 0.1% HgCl_2_ for 10 min, then rinsed in distilled water 10 times and soaked in the dark overnight at room temperature. Then the seeds were placed on filter papers soaked with distilled water until germination. The germinated seeds were transferred into trays (one seedling per well) with their roots soaked into the Hoagland solution (solution was changed every 2–3 days). When the seedling went to the three-leaf stage, 20% polyethylene glycol 6000 (PEG6000, Sigma Aldrich), 100 μM abscisic acid (ABA, Sigma Aldrich) and 200 mM NaCl (Sigma Aldrich) were added to simulate drought and salt stress. Roots were collected at 0, 1, 2 and 6 h after PEG6000 treatment, and at 0, 1, 3, 6, 12 and 24 h after ABA and NaCl treatment. Every treatment had three groups, each group had 3 duplicates. Root was immediately frozen in liquid nitrogen, total RNAs were extracted with TRIzol method.

### Semi-quantitative RT-PCR

Total RNAs were extracted from different tissues, different developmental stages or roots under different stress conditions by TRIzol method. Synthesis of cDNAs was performed with 2 μg of total RNAs, 50 ng of random hexamer primers (for ncRNAs) or oligo d(T)15 (for protein coding genes) were used in reverse transcription with the first strand cDNA synthesis kit (Promega). PCR primers were designed using VectorNTI software (Thermo Fisher Scientific). The primers used for ncRNAs and protein coding genes are listed in Additional file 2: Table S4 (for Quantitative real-time PCR) and Table S5 (for Semi-quantitative PCR). Semi-quantitative PCR was performed at an annealing temperature of 55 °C or 58 °C. U6 and tubulin were used as internal controls for ncRNAs and protein coding genes, respectively.

## Additional files

Additional file 1: Information of all novel ncRNAs that being identified in maize inbred line Chang 7–2. (XLSX 35 kb)

Additional file 2: **Figure S1.** Functional class distribution of host genes of intron-origin ncRNAs. **Figure S2.** Expression of UTR-origin ncRNAs (A and B) and their host genes (C) as well as ORF-origin U1 snRNAs (D) in different tissues of maize. **Figure S3.** Functional class distribution of protein-coding genes of UTR and ORF-origin ncRNAs. **Figure S4.** Genome location of ncRNAs and their neighboring genes. **Figure S5.** Expression of intron-origin ncRNAs and their host genes in different tissues of maize. **Figure S6.** Expression of intergenic-origin ncRNAs (A) and their neighboring genes (B) in different tissues of maize. **Table S1.** Genome location of novel ncRNAs of maize. **Table S2.** Probe sets used for removal of known ncRNAs like rRNAs and U snRNAs. **Table S3.** Primer sets for 5′ and 3’ RACE. **Table S4.** Primer sets for real-time PCR. **Table S5.** Primer sets for Semi-quantitative RT-PCR. (PDF 409 kb)

## Abbreviations

5-HT2CR: Serotonin (5-HT) 2C Receptor
ABA: Abscisic acid
DAG: Day after germination
DAP: Days after pollination
FLC: FLOWERING LOCUS C
miRNA: microRNA
ncRNAs: Non-coding RNAs
ORFs: Open Reading Frames
PEG: Polyethylene glycol
piRNA: Piwi-interacting RNA
PRC2: Polycomb repressive complex 2
siRNA: Small interfering RNA
snoRNA: Small nucleolar RNA
snRNA: Small nuclear RNA
SRP: Signal recognition particle
TE-lincRNAs: Transposable-element-origin long intergenic non-coding RNAs
UTR: Untranslated region
