# Supplementary material for: Identification and functional characterization of intermediate-size non-coding RNAs in maize
Source: BMC Genomics. 2018 Oct 4;19:730. doi: 10.1186/s12864-018-5103-1 (PMC6172812; doi:10.1186/s12864-018-5103-1)
Supplement: Supplementary file 2 — Figure S1. Functional class distribution of host genes of intron-origin ncRNAs. Figure S2. Expression of UTR-origin ncRNAs (A and B) and their host genes (C) as well as ORF-origin U1 snRNAs (D) in different tissues of maize. Figure S3. Functional class distribution of protein-coding genes of UTR and ORF-origin ncRNAs. Figure S4. Genome location of ncRNAs and their neighboring genes. Figure S5. Expression of intron-origin ncRNAs and their host genes in different tissues of maize. Figure S6. Expression of intergenic-origin ncRNAs (A) and their neighboring genes (B) in different tissues of maize. Table S1. Genome location of novel ncRNAs of maize. Table S2. Probe sets used for removal of known ncRNAs like rRNAs and U snRNAs. Table S3. Primer sets for 5′ and 3’ RACE. Table S4. Primer sets for real-time PCR. Table S5. Primer sets for Semi-quantitative RT-PCR. (PDF 409 kb) [file 12864_2018_5103_MOESM2_ESM.pdf]

Additional file 1:

Identification and functional characterization of intermediate-size non-coding RNAs in maize

Dandan Li, Huili Qiao, Wujie Qiu, Xin Xu, Tiemei Liu, Qianling Jiang, Renyi Liu, Zhujin Jiao, Kun Zhang, Lijun Bi, Runsheng Chen, Yunchao Kan

1. Figure S1. Functional class distribution of host genes of intron-origin ncRNAs

2. Figure S2. Expression of UTR-origin ncRNAs and their host genes as well as ORF-origin U1 snRNAs in different tissues of maize

3. Figure S3. Functional class distribution of protein-coding genes of UTR and ORF-origin ncRNAs

4. Figure S4. Genome location of ncRNAs and their neighboring genes

5. Figure S5. Expression of intron-origin ncRNAs and their host genes in different tissues of maize

6. Figure S6. Expression of intergenic-origin ncRNAs and their neighboring genes in different tissues of maize

7. Table S1. Genome location of novel ncRNAs of maize

8. Table S2. Probe sets used for removal of known ncRNAs like rRNAs and U snRNAs

9. Table S3. Primer sets for 5' and 3' RACE

10. Table S4. Primer sets for real-time PCR

11. Table S5. Primer sets for Semi-quantitative RT-PCR

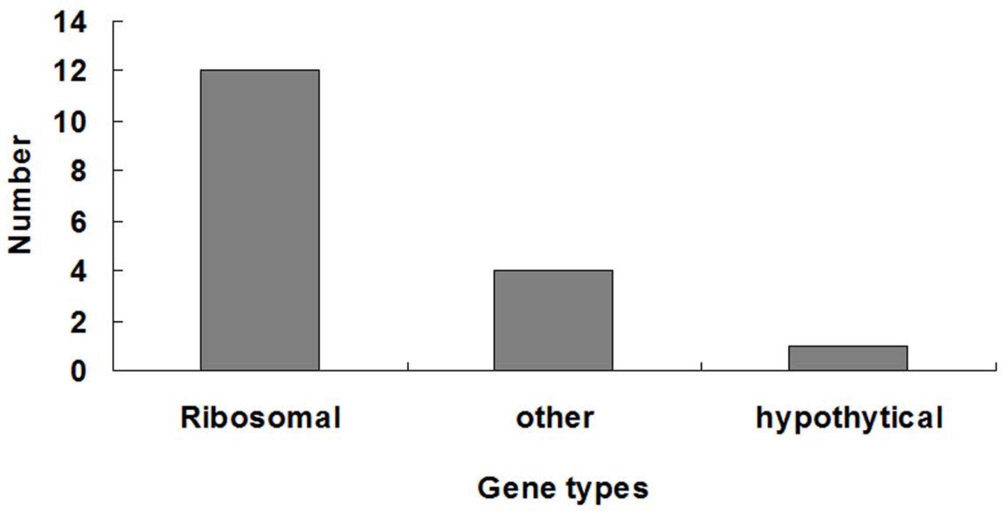

Figure S1. Functional class distribution of host genes of intron-origin ncRNAs. Other means genes with various functions.

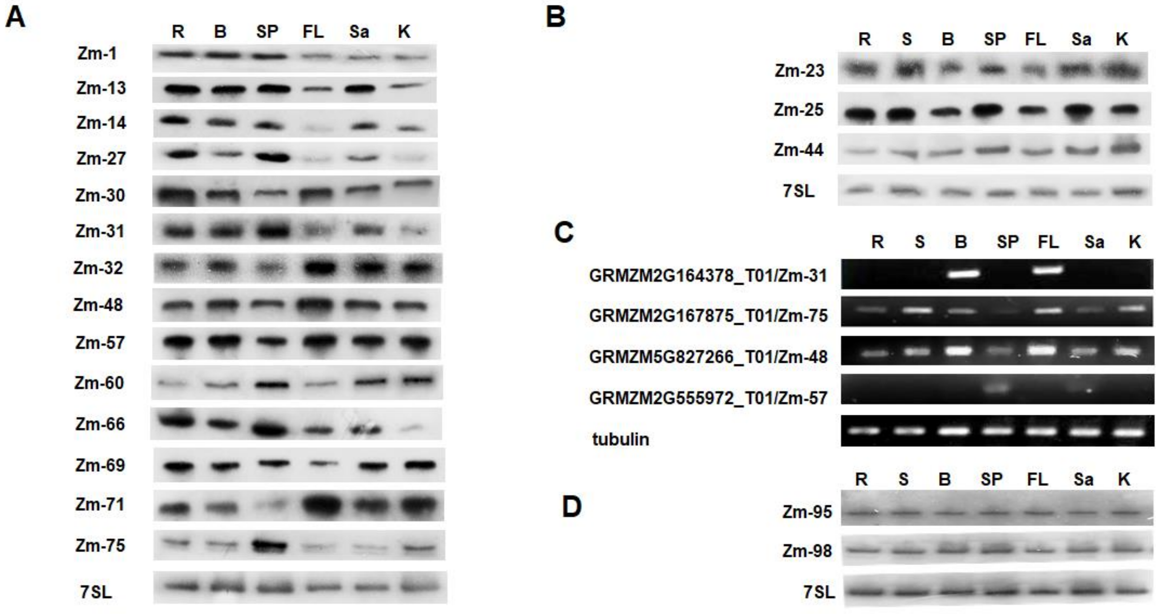

Figure S2. Expression of UTR-origin ncRNAs (A and B) and their host genes (C) as well as ORF-origin U1 snRNAs (D) in different tissues of maize.

R, S, B, SP, FL, Sa, H and K represent root, stem, blade, sheath & petiole, flag leaf, stem apex, hypocotyl and developing kernel, respectively. 7SL was used as an internal control in (*A*) (*B*) and(*D*), the *tubulin* gene was used as an internal control in (*C*).

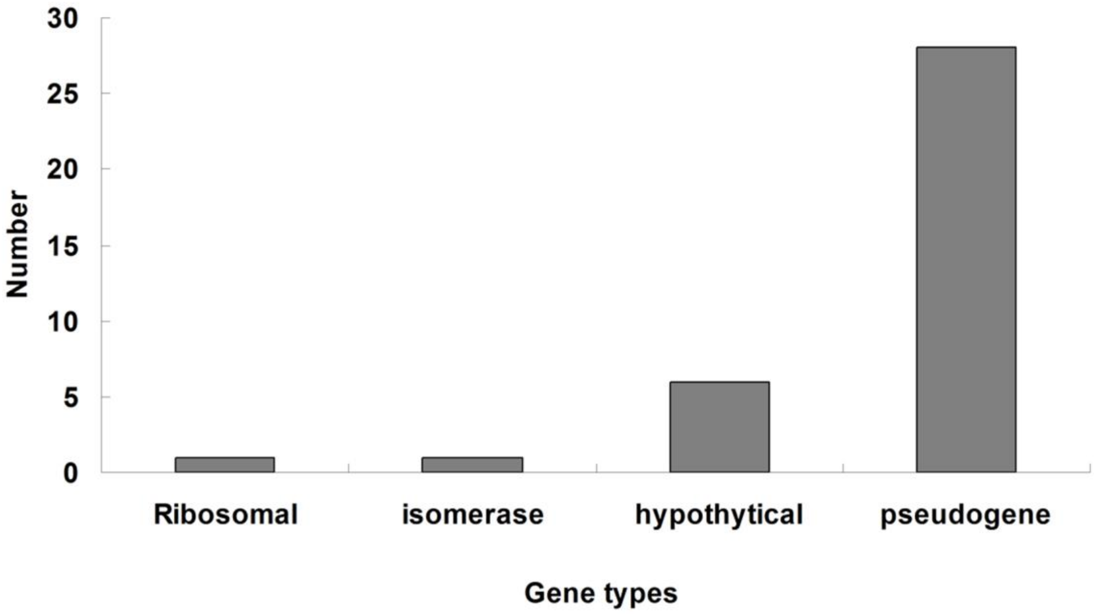

Figure S3. Functional class distribution of host genes of UTR and ORF-origin ncRNAs.

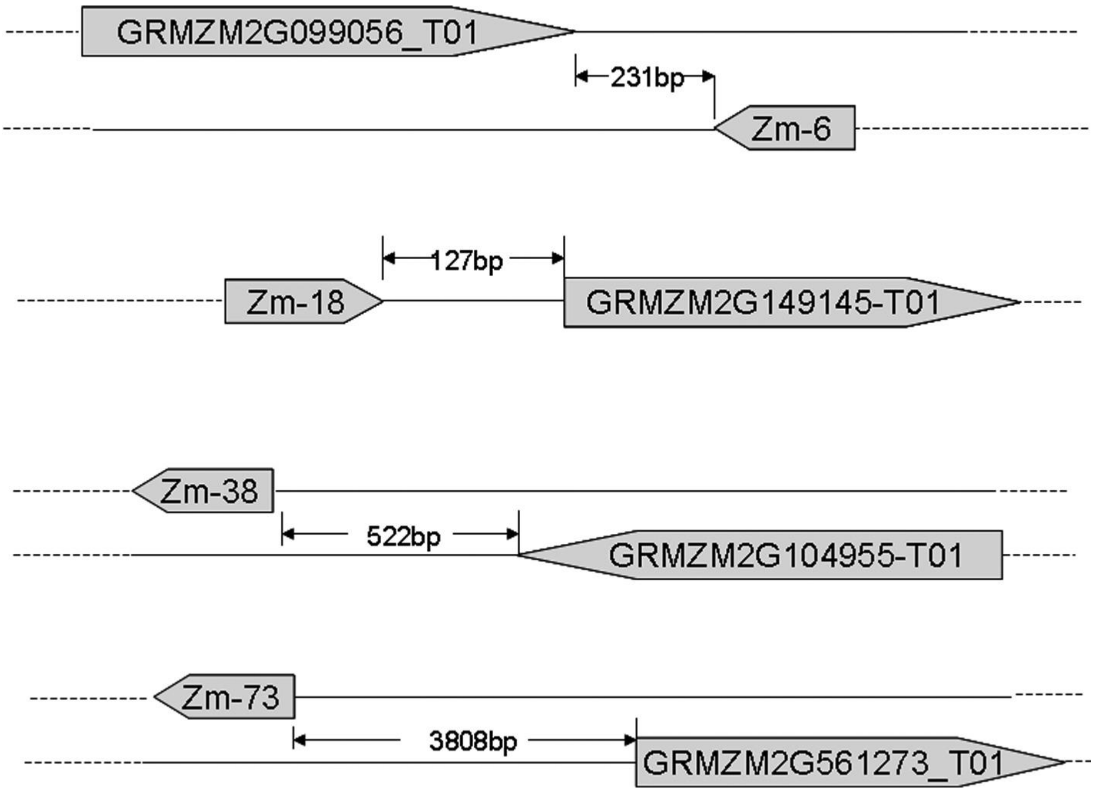

Figure S4. Genome location of ncRNAs and their neighboring genes.

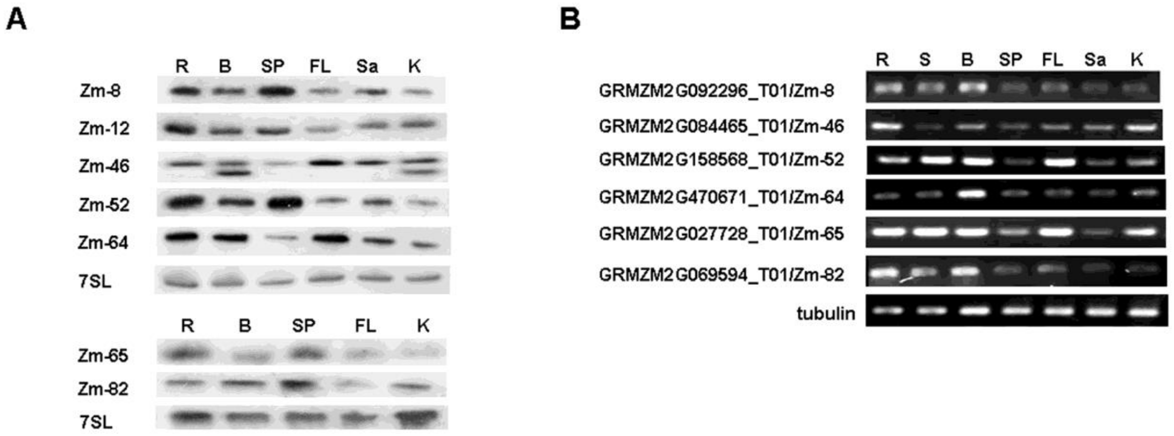

Figure S5. Expression of intron-origin ncRNAs and their host genes in different tissues of maize.

R, S, B, SP, FL, Sa, H and K represent root, stem, blade, sheath & petiole, flag leaf, stem apex, hypocotyl and developing kernel, respectively. 7SL was used as an internal control in (*A*), the *tubulin* gene was used as an internal control in (*B*).

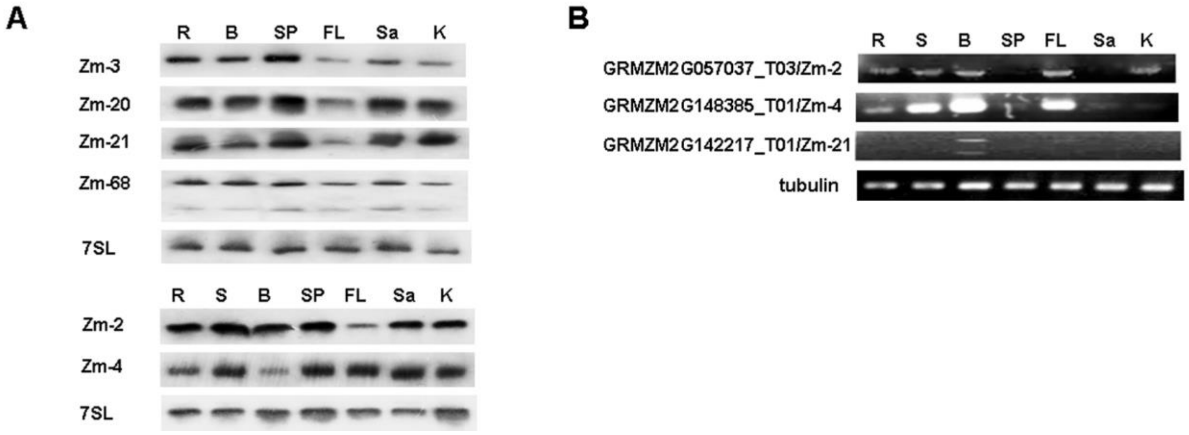

Figure S6. Expression of intergenic-origin ncRNAs (A) and their neighboring genes (B) in different tissues of maize.

R, S, B, SP, FL, Sa, H and K represent root, stem, blade, sheath & petiole, flag leaf, stem apex, hypocotyl and developing kernel, respectively. 7SL was used as an internal control in (*A*), the *tubulin* gene was used as an internal control in (*B*).

Table S1 Genome location of novel ncRNAs of maize

Genomic location	*Novel (%)*
Within transcribed sequence (sense)	***54 (48.65)***
Intron	***17 (15.32)***
In ORF	***9 (8.11)***
In UTR	***25 (22.60)***
Overlapping exon and intron (Zm-107)	***1 (0.90)***
Overlapping UTR and ORF	***2 (1.80)***
Antisense to transcribed sequence	***20 (18.02)***
to intron	***2 (1.80)***
to exon	***7 (6.31)***
to UTR	***10 (9.00)***
Overlapping UTR and ORF(Zm-29)	***1 (0.90)***
Intergenic	***37 (33.33)***
All	***111***

Table S2 Probe sets used for removal of known ncRNAs like rRNAs and U snRNAs

Name	*Sequence*
5S-1	AAAAAAAAAAAAAAAAAAAACATATATATCGAGGTCGGAA
5S-2	AAAAAAAAAAAAAAAAAAAATATATAACGAGGTCGGAATG
5S-3	AAAAAAAAAAAAAAAAAAAACGTCACCGCAGTAGAGTTTA
5S-4	AAAAAAAAAAAAAAAAAAAAGGGATGGATTGGTGTGGTTC
5S-5	AAAAAAAAAAAAAAAAAAAATACTACTCTCGCCCAAGCAC
5S-6	AAAAAAAAAAAAAAAAAAAACTGCGGAGTTCTGATGGGAT
5.8S-1	AAAAAAAAAAAAAAAAAAAAGTGACGCCCTCCGCCAGAAG
4.5S-1	AAAAAAAAAAAAAAAAAAAAAATCGTTCGTTCGTTAGGA
4.5S-2	AAAAAAAAAAAAAAAAAAAACACAGACCTTCCACTTGACA
28S-1	AAAAAAAAAAAAAAAAAAAAAGTTCTGAGTCGGTTGTTCG
18S-1	AAAAAAAAAAAAAAAAAAAATACTACGCAGGCTCATCAAA
18S-2	AAAAAAAAAAAAAAAAAAAAACTTTCACTTTCAACCCGAT
18S-3	AAAAAAAAAAAAAAAAAAAAGGGTATCTAATCCTGTTCGC
18S-4	AAAAAAAAAAAAAAAAAAAATATGTCAAGGGCTGGTAAGG
18S-5	AAAAAAAAAAAAAAAAAAAATAACCAAGCCTAACCAAACC
18S-6	AAAAAAAAAAAAAAAAAAAAGAGAACAATCCGAACTGAGG
25S-1	AAAAAAAAAAAAAAAAAAAAGCCTGTATTTAGCCTTGGAC
25S-2	AAAAAAAAAAAAAAAAAAAAGCTCAGGCATAGTTCACCAT
25S-3	AAAAAAAAAAAAAAAAAAAAACCATCGTCCTGCTGTCTTA
25S-4	AAAAAAAAAAAAAAAAAAAAGCTCAACAGGGTCTTCTTTC
25S-5	AAAAAAAAAAAAAAAAAAAACAATGATAGGAAGAGCCGAC
U2-1	AAAAAAAAAAAAAAAAAAAACTAGAGCGACACATAAGGGA
U2-2	AAAAAAAAAAAAAAAAAAAACGATAGCGACACATAAGGGA
U5-1	AAAAAAAAAAAAAAAAAAAATAGGCGTATGCTGCTTGTTG

U6-1	AAAAAAAAAAAAAAAAAAAAAATTTGTGCGTGTCATCCTTG
U3-1	AAAAAAAAAAAAAAAAAAAAAACTCCAGACAACACACCAAGG
U3-2	AAAAAAAAAAAAAAAAAAAAAAGTTAATCATGCTCT

Table S3 Primer sets for 5' and 3'RACE

3'RACE	***Primers***
Zm-5	TGATGAGAAATACCTCCTAC
Zm-42	GATGATGAAATTGCTCCGTG
Zm-47	ATTTGGTATCTGACTCGCAG
Zm-52	TCGGGTATAGTCAGGTGTCT
Zm-83	AGGAGGAGCACTAGTTTACC
Zm-95	CGACGTGGGATCAAGAAGCC
Zm-102	TGGCTTGCCACTGGGTTCCT
Zm-103	TTGCCACTGGGTTCCTCAAG
Zm-104	TCCTCGGTAGTATAGTGTAC
Zm-105	CCGTTGCACTACTGGCTGAG
Zm-107	CTTGCTGCTGGGGTCCTCAT
U2-1	TTTTGTGGGGGAGGGACCAC
U2-2	GAGGGACCACAACAGTGGCT
U5-3	GCAAATAGCAGCATACGCCT
5CD	GGAGTAGCATGCGTGACGAAA
5'RACE	
Zm-5	TCAGAATGAGTAGGAG
Zm-42	GTGCTCTTCTATCACCTGAG
Zm-47	AACTGGAAGCTCATAAATG
Zm-52	TCGGGTATAGTCAGGTGTCT
Zm-83	GTATCCCCTCTGCCACAAAT
Zm-95	CTGTGAGCTCTGCAATGCAGGCCAC
Zm-102	CTCAGCCTGTAGTGCAAC
Zm-103	GTCCAGCCAATAGTGCAAC
Zm-104	CTCAGCCTGTAGTGCAAC
Zm-105	TATGAGCTCGCTCAGCCAGTAGT
Zm-107	TTGTGAGCTCGGCTTGGGCAGTAGT
U2-1	TGTGAGCTCCTCAGCCTGTAGTG
U2-2	ATAGAGCTCCTCAGCCTGTAGTGC
U5-3	GTCCTCGCAGAGAGAAAC
3RT	CCCTGTGAGCTCGTGGTCAA

Table S4 Primer sets for real-time PCR

ncRNAs	***Primers***
Zm-6-F	CAGCAGTGATAGTAAAAGAATGACT

Zm-6-R	GCAGCTCAACAGTGCTTGAA
Zm-18-F	AAACGATGATGAAAAGCTCTG
Zm-18-R	AAGCTCAGAGGTGCTCGTAT
Zm-73-F	ACTAAAACAATCAGGCTTGAT
Zm-73-R	AAAAAGATGCATTTAAGAGG
Neighboring genes	
GRMZM2G166424_T01-F	ATGCCCAGGCCAAGTGTAGC
GRMZM2G166424_T01-R	TAGCCCGTATCCCTGCCTTG
GRMZM2G099056_T01-F	ATCTTCGAGAAGCTCGGCAG
GRMZM2G099056_T01-R	CCTTTGCCCAAAGAGCTAGC
GRMZM2G149145_T01-F	CTGCAGCCTCCACTGTTGCT
GRMZM2G149145_T01-R	CCTGTTTTGTCAGCCACCAT
GRMZM2G104955_T01-F	GGTGGCGCGTTCTCTTCTTC
GRMZM2G104955_T01-R	CTGTCCAGGGATTCATCGCC
GRMZM2G084465_T01-F	AAGCTCAAGACCCACCGCAG
GRMZM2G084465_T01-F	CGGAAACCTTGACGACCTTG
GRMZM2G561273_T01-F	ATGGATCCCAACGACGCCTT
GRMZM2G561273_T01-R	TCCAATGTGCTCCCGGTGTG

Table S5 Primer sets for Semi-quantitative RT-PCR

Names of up-downstream and neighboring genes	***Primers***
GRMZM2G057037_T03-F	GCTGGTGCCTGGAGTGGAAT
GRMZM2G057037_T03-R	CACGGATGCCTGTGCCATAG
GRMZM2G148385_T01-F	CCGCCGTCATCGTTCCTTTT
GRMZM2G148385_T01-R	GCCAGCACGACCCACAAAAC
GRMZM2G092296_T01-F	ATGATGGATCCGCAGCACAG
GRMZM2G092296_T01-R	ACCTCAACCTCGACACCTGG
GRMZM2G142217_T01-F	ATGCCTTTTCTTCACGTCGG
GRMZM2G142217_T01-R	GTTCATCATCCACGGCCATT
GRMZM2G164378_T01-F	CACGCAGGTCCAGCTAGCTCTA
GRMZM2G164378_T01-R	GCGTGAGATTCCCTGTGACG
GRMZM5G827266_T01-F	GAAAAACTCAGCACAAACATTC
GRMZM5G827266_T01-R	AGGAAGCATGCGTCTATGAT
GRMZM2G158568_T01-F	ATGGCGGAGAAGAAGCGAGG
GRMZM2G158568_T01-R	CCGGTGGGATCTCAGCAACT

GRMZM2G555972_T01-F	ATTCCGTCTCCTAGTTTTGG
GRMZM2G555972_T01-R	CATCTTCATCACAGCACCAT
GRMZM2G470671_T01-F	ATGCCATTTGGTACGAGTAG
GRMZM2G470671_T01-R	AGGGTTAAAAGTTTCACGGT
GRMZM2G027728_T01-F	ATGGTGGCCACAAAGAAGAC
GRMZM2G027728_T01-R	GCAGCAGACCCGAAAGTATT
GRMZM2G167915_T01-F	ATGAGCACCCCAAGATCTAC
GRMZM2G167915_T01-R	TAAAGTTGATTGTTGCCGTC
GRMZM2G069594_T01-F	ATGCCGAAGCAGATCCACGA
GRMZM2G069594_T01-F	CCTGGGGGGAGAGATTGCTT
